# Lack of Oncomodulin Increases ATP-Dependent Calcium Signaling and Susceptibility to Noise in Adult Mice

**DOI:** 10.1523/ENEURO.0142-26.2026

**Published:** 2026-06-02

**Authors:** Yang Yang, Jing-Yi Jeng, Kaitlin Murtha, Leslie K. Climer, Federico Ceriani, Weintari D. Sese, Aubrey J. Hornak, Kiah C. Sleiman, Ian Stahl, M. Charles Liberman, Walter Marcotti, Dwayne D. Simmons

**Affiliations:** ^1^Department of Biology, Baylor University, Waco, Texas 76798; ^2^School of Biosciences, University of Sheffield, Sheffield S10 2TN, United Kingdom; ^3^Department of Otolaryngology, Head & Neck Surgery, Harvard Medical School, Boston, Massachusetts 02114; ^4^Eaton-Peabody Laboratories, Mass Eye and Ear, Boston, Massachusetts 02114; ^5^Sheffield Neuroscience Institute, University of Sheffield, Sheffield S10 2TN, United Kingdom; ^6^Department of Integrative Biology and Physiology, University of California, Los Angeles, California 90095

**Keywords:** EF-hand calcium-binding protein (CaBP), GCaMP6s, noise-induced hearing loss, oncomodulin (OCM), outer hair cell (OHC), purinergic receptor

## Abstract

Dysregulation of Ca^2+^ homeostasis in cochlear outer hair cells (OHCs) is associated with impaired hearing following noise exposure. Ca^2+^ signaling in developing OHCs is modulated by oncomodulin (OCM), an EF-hand calcium-binding protein. Here, we investigated whether the lack of OCM disrupts Ca^2+^ signaling in mature OHCs and influences vulnerability to moderate noise. Using young adult CBA/CaJ mice of either sex, we found that OHCs from *Ocm* knock-out (*Ocm^−/−^*) mice showed comparable electromotile responses and synaptic innervation compared with littermate controls. Prior to noise exposure, *Ocm*^−/−^ mice had auditory brainstem responses with highly variable latencies and amplitudes compared with *Ocm*^+/+^ mice. Moderate noise exposure (95 dB SPL, 2 h) caused temporary threshold shifts in wild-type (*Ocm*^+/+^) mice but PTS in *Ocm*^−/−^ mice. Using a genetically encoded Ca^2+^ sensor (GCaMP6s) expressed in OHCs, we found increased GCaMP6s fluorescence and ATP-induced Ca^2+^ signaling in *Ocm*^−/−^ OHCs. Using GCaMP6s mice of either sex, P2X2 expression was also higher throughout the cochlea of *Ocm*^−/−^ mice compared with *Ocm*^+/+^ mice. Prolonged noise exposure (95 dB SPL, 9 h) led to greater threshold shifts in *Ocm*^−/−^ mice and upregulated expression of P2X2 receptors in the cochlea of *Ocm*^+/+^ but not in the *Ocm*^−/−^ mice. Prolonged noise exposure did not change the number of presynaptic OHC ribbons. We propose that the lack of OCM leads to a noise-exposed phenotype that increases susceptibility to cochlear pathology. Additionally, the combination of increased purinergic signaling and dysregulation of cytosolic Ca^2+^ homeostasis likely contributes to early-onset hearing loss in *Ocm*^−/−^ mice.

## Graphical abstract

**Visual Abstract:**
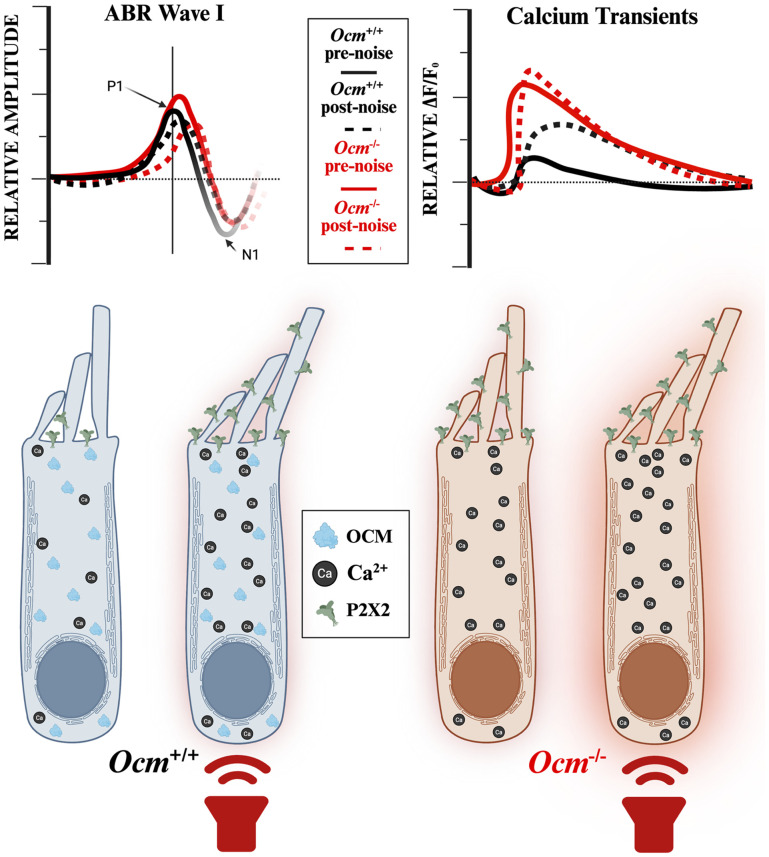
Schematic representing altered ABR waveforms and Ca^2+^ transients in *Ocm*^−/−^ and *Ocm*^+/+^ OHCs with moderate (95 dB SPL) noise exposure. In *Ocm*^+/+^ mice, moderate noise induces slight latency and amplitude shifts, increased purinergic receptor expression, and increased Ca^2+^ signaling. *Ocm*^−/−^ OHCs exhibit elevated Ca^2+^ signaling prenoise exposure and exhibit more dramatic latency and amplitude shifts postnoise exposure with no significant change in purinergic receptor expression after moderate noise exposure.

## Significance Statement

This study shows that loss of a cytosolic calcium buffer, oncomodulin (OCM), produces an adult cochlea that functionally resembles a noise-exposed ear and is significantly more vulnerable to noise-induced hearing loss. Before noise exposure, OCM deficiency in outer hair cells (OHCs) modifies auditory-nerve responses, heightens ATP-evoked calcium responses, and elevates purinergic receptor expression, which likely leads to increased susceptibility to noise damage. After moderate noise exposure, OCM-deficient mice exhibit larger, permanent auditory threshold shifts. The findings indicate that OCM is essential for OHC function but also for protecting the cochlea from acoustic stress in the adult.

## Introduction

In response to sound stimulation, Ca^2+^ influx and Ca^2+^-induced Ca^2+^ release from the endoplasmic reticulum significantly increase intracellular Ca^2+^ in cochlear sensory cells. Factors that cause hearing impairment, such as ototoxic drugs, acoustic overstimulation, and aging, are associated with dysregulation of Ca^2+^ homeostasis (Fridberger and Ulfendahl, 1996; Liu and Fechter, 1997; Kidd Iii and Bao, 2012; Richard et al., 2023). Two types of specialized sensory cells in the mammalian cochlea, inner hair cells (IHCs) and outer hair cells (OHCs), are involved in the transduction of sound into electrical responses (Dallos, 1992). The OHCs are largely responsible for the sensitivity and tuning, whereas the IHCs transmit auditory signals to the brain via the auditory nerve. One of the hallmarks of cochlear injury following noise exposure is loss of OHCs. However, comparatively little is known about the endogenous mechanisms necessary to protect OHCs from the damaging effects of noise exposure. Shortly after acoustic overstimulation, intracellular Ca^2+^ concentration is increased in auditory hair cells, especially OHCs (Fridberger et al., 1998; Zuo et al., 2008), which leads to their loss (Fridberger et al., 1998; Orrenius et al., 2003; Esterberg et al., 2013). Thus, effective regulation of Ca^2+^ is essential for the responses of OHCs to acoustic overexposure.

Although evidence strongly suggests that Ca^2+^ overload can trigger apoptotic or necrotic cell death and thus contributes to noise-induced damage, molecular models for Ca^2+^ overload are lacking. In OHCs, Ca^2+^ homeostasis depends on the balance of Ca^2+^ influx, extrusion, and buffering. Oncomodulin (OCM) is a small, 12 kDa, EF-hand calcium-binding protein (CaBP) that is predominantly expressed in OHCs and functions differently from other EF-hand CaBPs (Pangrsic et al., 2015; Climer et al., 2019; Murtha et al., 2022). OCM is the β isoform of parvalbumin and shares at least 53% sequence identity with α-parvalbumin (PVALB). In adult mice, targeted deletion of OCM (*Ocm*^−/−^) is associated with early progressive hearing loss and, to date, is the only EF-hand CaBP in OHCs associated with hearing loss. The earlier onset of age-related threshold shifts *Ocm*^−/−^ mice is independent of genetic background, which indicates the importance of OCM in the long-term maintenance of OHC function (Tong et al., 2016; Climer et al., 2021). Recent studies have shown that the absence of OCM increases Ca^2+^ signaling in developing OHCs and reveal that OCM plays a significant role in the synchronization of Ca^2+^ activity during cochlear maturation (Murtha et al., 2022; Yang et al., 2023). Additionally, the onset of OCM expression alters the level of Ca^2+^ signals in OHCs before hearing onset (Murtha et al., 2022). Recent findings also reveal higher levels of spontaneous Ca^2+^ activity, upregulation of purinergic receptors in OHCs, and higher synchronization of Ca^2+^ activity in prehearing *Ocm*^−/−^ mice compared with wild-type mice (Yang et al., 2023). Given that OCM can modulate Ca^2+^ signaling and synchronization in OHCs during the prehearing period and is essential for maintaining hearing in young adults, the present study hypothesized that the lack of OCM in adult mice increases OHC vulnerability to noise.

A recent study suggests that *Ocm* deletion leads to mitochondrial changes and OHC loss after exposure to high-level, broadband noise (Murtha et al., 2024). In the present study, we used young adult *Ocm*^−/−^ and age-matched controls to study the underlying mechanisms that may contribute to increased susceptibility to permanent cochlear threshold shifts. Two different noise exposure paradigms were used to probe for differences in hearing recovery and cumulative Ca^2+^ response. Prior to noise exposure, adult *Ocm*^−/−^ mice demonstrated a noise exposure phenotype with more variable amplitude and latency responses and upregulated purinergic receptor expression. In response to moderate noise exposures, *Ocm*^−/−^ mice showed larger threshold shifts and little change in P2X2 expression compared with *Ocm*^+/+^ mice. We conclude that the increased vulnerability to noise in *Ocm*^−/−^ mice may be associated with preexisting, elevated levels of both Ca^2+^ signaling and purinergic receptor expression and ultimately leads to an early onset of age-related hearing loss as observed in other reports (Tong et al., 2016; Climer et al., 2021).

## Materials and Methods

### Ethical approval

All procedures performed were approved by the authors’ university Institutional Animal Care and Use Committees as established by the US Public Health Service and performed in compliance with the National Institutes of Health animal care guidelines and/or licensed by the UK Home Office under the Animals (Scientific Procedures) Act 1986.

### Animals

Animals were bred in animal facilities at the authors’ facilities. The *Ocm* mutant mouse (C57Bl/6 *ActbCre*;*Ocm^flox/flox^*) was backcrossed onto the CBA/CaJ and the CBA/CaH background to minimize or eliminate any confounding effects of *Cdh23*^ahl^ mutation, which is linked to hearing loss in the adult (Liu et al., 2007).

We also generated mice with a genetically encoded, tissue-specific Ca^2+^ sensor (*Atoh1*-GCaMP6s) in *Ocm* wild-type (GCaMP6s-*Ocm*^+/+^) and *Ocm* knock-out (GCaMPs-*Ocm*^−/−^) mice. We first crossed *Ocm* wild-type (*Ocm^+/+^*) and *Ocm* knock-out (*Ocm^−/−^*) mice with B6;129S-*Gt(ROSA)26Sor^tm96.1(CAG-GCaMP6s)Hze^*/J, Ai96(RCL-GCaMP6s) or Ai96, which contains a floxed-STOP cassette preventing transcription of the GCaMP6 slow variant Ca^2+^ indicator. These mice were then crossed with knock-in transgenic mice expressing Cre recombined from the *Atoh1* locus (Chen et al., 2013). *Atoh1*-driven Cre GCaMP6s mice showed tissue-specific expression of endogenous green fluorescence (Yang et al., 2010; Cox et al., 2012; Mulvaney and Dabdoub, 2012).

### Single-cell electrophysiology

For electrophysiological recordings, OHCs from CBA/CaH control (*Ocm^+/−^*) and *Ocm* knock-out (*Ocm^−/−^*) mice of both sexes were acutely dissected at 1–2 months. Cochleae were isolated from the inner ear using an extracellular solution composed of the following (in mM): 131 NaCl, 5.8 KCl, 1.3 CaCl_2_, 0.9 MgCl_2_, 0.7 NaH_2_PO_4_, 5.6 d-glucose, and 10 HEPES-NaOH. Sodium pyruvate (2 mM), amino acids, and vitamins (Thermo Fisher Scientific). The pH was adjusted to 7.5 (osmolality ∼308 mmol kg^−1^). The dissected cochleae were fixed at the bottom of the recording chamber by a nylon-meshed silver ring and perfused with the above extracellular solution. OHCs were viewed using an upright microscope (Olympus BX51) equipped with Nomarski differential interface contrast optics with a 60× water immersion objective and 15× eyepieces.

Recordings were performed at room temperature (21–24°C) using an Optopatch amplifier (Cairn Research). Patch pipettes were pulled from soda glass capillaries, and the shank of the electrode was coated with surf wax (2–3 MΩ). Current and voltage responses were measured using the following intracellular solution (in mM): 145 KCl, 3 MgCl_2_, 1 EGTA-KOH, 5 Na_2_ATP, 5 HEPES-KOH, 10 sodium phosphocreatine, pH adjusted to 7.28 with KOH (osmolality was 294 mmol kg^−1^). Voltage-clamp protocols were performed at a holding potential of −84 mV. Data acquisition was performed using pClamp software (Axon Instruments) using Digidata. Data were filtered at 2.5 kHz (eight-pole Bessel), sampled at 5 kHz, and stored on the computer. Offline data analysis was performed using Origin software (OriginLab). Membrane potentials reported were corrected for the uncompensated residual series resistance (*R*_s_) and the liquid junction potential, which was −4 mV, measured between electrode and bath solutions.

### Electromotile response

Electromotility was estimated in OHCs at room temperature (∼22°C) by applying a depolarizing voltage step from the holding potential of −64 to +56 mV and recorded using a CCD camera (Thorlabs DCU224M). The camera was attached to a microscope (Olympus), equipped with a 60× water immersion objective (Olympus LUMPlanFL N). The acquired images were stack-sliced along a vertical axis of each OHC, and the contraction was measured on the image stack as length change of the cell. All images were analyzed in ImageJ, and the measurements were calibrated using a stage graticule.

### Cochlear function assays

For measurement of auditory brainstem responses (ABRs) and distortion product otoacoustic emissions (DPOAEs), adult mice were anesthetized with xylazine (20 mg/kg, i.p.) and ketamine (100 mg/kg, i.p.). Acoustic stimuli were delivered using a custom acoustic assembly (Maison et al., 2012). Briefly, two electrostatic earphones (CUI CDMG15008-03A) were used to generate primary tones and a Knowles miniature microphone (EK-3103) was used to record ear-canal sound pressure. Stimuli were generated digitally with 4 ms sampling. Ear-canal sound pressure and electrode voltage were amplified and digitally sampled at 20 ms for analysis of response amplitudes. The acoustic stimuli and physiological responses were digitized by a National Instruments PXI system with 24 bit sound cards running custom LabVIEW software. ABRs were recorded via 30 gauge platinum electrodes, inserted subdermally, adjacent to the pinna incision and at the vertex of the skull, with a ground near the tail. Tone-pip stimuli were presented at half-octave frequency intervals from 5.6 to 45.2 kHz. At each frequency, a level series was presented, in either 5 or 10 dB steps, from below threshold to 80 dB SPL, with up to 1,024 stimuli averaged per step. Sound-evoked responses were amplified 10,000× through a 0.3–3 kHz bandpass filter. ABR threshold was defined as the lowest stimulus level to generate a coherent set of response peaks that increased in amplitude and decreased in latency as the stimulus level increased. The amplitude and latencies of the ABR waveform potentials were also measured for wave I (P1–N1; Ceriani, 2024). For measurement of DPOAEs at 2*f*_1_–*f*_2_, the primary tones were set so that the frequency ratio (*f*_2_/*f*_1_) was 1.2 and so that the *f*_2_ level was 10 dB below the *f*_1_ level. For each *f*_2_/*f*_1_ primary pair, levels were swept in 5 or 10 dB steps from 10 dB SPL to 80 dB SPL (for *f*_2_). At each level, both waveform and spectral averaging were used to increase the signal-to-noise ratio of the recorded ear-canal sound pressure, and the amplitude of the DPOAE at 2*f*_1_–*f*_2_ was extracted from the averaged spectra, along with the noise floor at nearby points in the spectrum. Iso-response curves were interpolated from plots of DPOAE amplitude versus sound level. The threshold was defined as the *f*_2_ level required to produce a DPOAE at 0 dB SPL. Right ears were used for all hearing tests. For the total threshold shift of ABR and DPOAE, the total threshold shifts across all frequencies for each animal were calculated and then averaged across all animals.

### Noise exposure

Two different experimental approaches were used to assess the effects of noise exposure. The first approach assessed temporary and permanent threshold shifts (PTS) after short duration (2 h) noise exposure, while the second approach assessed the immediate effects of sustained (9 h) noise exposure. The 4–6-week-old *Ocm*^+/+^ and *Ocm*^−/−^ CBA/CaJ mice and 3–4-week-old GCaMP6s *Ocm*^+/+^ and *Ocm*^−/−^ were randomly assigned to either noise-exposed or control groups. Cochlear thresholds of age-matched mice were measured 24 h prior to noise exposure. Unexposed mice served as controls. CBA/CaJ mice were exposed to an octave band noise for 2 h then cochlear function was assessed at 6 h and 2 weeks later, followed by cochlear fixation and tissue harvest. For GCaMP6s mice, exposure was to broadband noise at 95 dB SPL for 9 h. Cochlear function was assessed 1 h following noise exposure followed by tissue harvesting.

For both groups, awake and unrestrained mice were placed in individual sections of a wire-mesh cage and exposed to either octave band noise (8–16 kHz) for 2 h at 95.0 dB SPL or broadband noise for 9 h at 95 dB SPL. The booth is equipped with a JBL2446H compression driver coupled to an exponential horn. Noise levels were calibrated and monitored either with a 1⁄4′′ Brüel and Kjær condenser microphone or a sound-level meter. The noise level varied by <2 dB over the duration of the exposures. Food and water were provided in a Petri dish during 9 h of noise exposure.

### Tissue preparation

Cochleae were harvested from 3–4-week-old *Ocm*^+/+^ or *Ocm*^−/−^ mice of either sex. After decapitation, apical coil OHCs were dissected from the organ of Corti in an extracellular medium composed of the following (in mM): 136.8 NaCl, 5.4 KCl, 0.4 KH_2_PO_4_, 0.3 Na_2_HPO_4_, 0.8 MgSO_4_, 1.3 CaCl_2_, 4.2 NaHCO_3_, 5 HEPES, and 5.6 glucose. The pH was adjusted to 7.4–7.5 (osmolality ∼306 mmol/kg). The apical coil was then transferred to a small microscope chamber with nylon mesh fixed to a stainless-steel ring on the bottom and visualized using an upright microscope (Leica, DM 6000 FS) with a water immersion objective (Leica).

### Confocal Ca^2+^ imaging

Ca^2+^ signals from GCaMP6s were recorded at room temperature with 480 nm excitation wavelength (X-Light V2 spinning disk confocal, 89 North, PRIME95B Photometrics Cooled sCMOS with 95% QE, Teledyne Photometric). Images were taken using VisiView (VISITRON) and analyzed offline using ImageJ (NIH). Ca^2+^ signals were measured as relative changes in fluorescence emission intensity (Δ*F*/*F*_0_) and calculated by MATLAB. Δ*F* = *F* − *F*_0_, where *F* is fluorescence at time *t* and *F*_0_ is the fluorescence at the onset of the recording.

For inducing Ca^2+^ transients, 100 µl of ATP solution was perfused into a microscope chamber containing 300 µl extracellular solution (1:4 dilution, 100 µM ATP final concentration in the chamber) at 8 s after the imaging started, followed by a 2 min perfusion with the extracellular solution to help with equilibration and wash out. Only the fluorescence signals from OHCs that reached maximum intensity between 8 and 60 s were calculated. For the P2X antagonist experiments, the blocker, PPADS (100 μM), was added to the explant right before ATP application. Each GCaMP6s fluorescence recording includes 1,500 frames taken at 15 fps from the 873 × 873 pixels region. After background subtraction, the time course of Ca^2+^ changes in activated OHCs was computed as pixel averages of a circle region of interest (ROI) using ImageJ.

### Genotyping and qRT-PCR

DNA was extracted from tail samples using Extract-N-Amp Tissue PCR Kit (Sigma-Aldrich). PCR primers used for genotyping are listed as follows: *Atoh1*-Cre primer pair forward, 5′-CCGGCAGAGTTTACAGAAGC-3′; reverse, 5′-ATG TTT AGC TGG CCC AAA TG-3′; Cre control primer pair forward, 5′-CTA GGC CAC AGA ATT GAA AGA TCT-3′; reverse, 5′-GTA GGT GGA AAT TCT AGC ATC ATC C-3′; GCaMP6s primer pair forward, 5′-ACG AGT CGG ATC TCC CTT TG-3′; reverse, 5′-AGA CTG CCT TGG GAA AAG CG-3′; *Ocm* primer pair forward, 5′-CTC CAC ACT TCA CCA AGC AG-3′, reverse, 5′-TTT CAT GTT CAG GGA TCA AGT G-3′; *Ocm* deletion primer pair forward, 5′-CTC CAC ACT TCA CCA AGC AG-3′; and reverse, 5′-GCT TGG GGA CCC CCT GTC TTC A-3′.

Cochleae were acutely dissected after anesthesia and transferred to the lysis buffer. Total RNA was extracted using RNeasy plus Micro kits (QIAGEN). iScript Advanced cDNA Synthesis Kit (Bio-Rad Laboratories) was used for reverse transcription.

qRT-PCR was performed using the SYBR Green PCR Master Mix Kit (Bio-Rad Laboratories) as previously described (Murtha et al., 2022). Briefly, the *b2m* gene was used as a reference gene (Melgar-Rojas et al., 2015). Quantification of expression (fold change) from the Cq data was calculated following the ΔΔCq method (Schmittgen and Livak, 2008) and normalized to the Cq value of *Ocm*^+/+^. To represent the relative expression (fold change), 2^−ΔΔCq^ was calculated. Primers used for qRT-PCR are as follows: *b2m* forward 5′-TGGTCTTTCTGGTGCTTGTC-3′ and reverse 5′-GGG TGG AAC TGT GTT ACG TAG-3′; *P2RX2* forward 5′-GCG TTC TGG GAC TAC GAG AC-3′ and reverse 5′-ACG TAC CAC ACG AAG TAA AGC-3′; *Ocm* primer pair forward, 5′-CTC CAC ACT TCA CCA AGC AG-3’; reverse, 5′-TTT CAT GTT CAG GGA TCA AGT G-3′ (PrimerBank ID 27544798a1).

### Immunofluorescence

Histological analysis and immunocytochemistry were performed. Briefly, cochleae were flushed with 4% PFA and then left in fixative overnight at 4°C with rotation. Cochleae were then decalcified in 0.1 M EDTA for 3–5 d at 4°C with rotation. The apical coils of cochleae were then dissected or prepared as 100 μm midmodiolar sections embedded in the gelatin-agarose solution (Maison et al., 2012). Microdissected cochlear pieces were suspended in 30% sucrose for 30–60 min at room temperature with gentle shaking, frozen at −80°C for 30 min, and then thawed for 30 min at 37°C. Pieces were washed three times in PBS and then blocked in 5% normal horse serum for 1 h at room temperature. Samples were labeled with antibodies to myosin 7A (Proteus Biosciences, number 25-6790, 1:200), the C-terminal-binding protein 2 (CtBP2, BD Biosciences #612044, 1:200), OCM (Santa Cruz Biotechnology, sc-7446, 1:200), GluR2 (Millipore, MAB397, 1:200), and P2X2 (Alomone Labs, APR-003, 1:400). Primary antibodies were incubated overnight at 37°C. Appropriate Northern Lights (R&D Systems 1:200) and Alexa Fluor (Invitrogen 1:200)-conjugated secondary antibodies were incubated for 1 h at 37°C. Slides were prepared using VECTASHIELD mounting media with DAPI (Vector Laboratories).

### Confocal microscopy

Images were acquired using a Zeiss LSM800 upright confocal laser scanning microscope (Zeiss). Cohorts of samples were immunostained at the same time and imaged under the same optical conditions to allow for direct comparison. Microdissected cochlear epithelia were imaged using a 10× air objective (N.A. 0.3) at 0.5× zoom to capture entire pieces. Then, higher-magnification images were taken using a 40× oil immersion objective (N.A. 1.2). *Z*-stacks were taken to capture whole hair cells, using Hoechst or DAPI, phalloidin, and Myo7a. A cochlear frequency map was constructed from these images by tracing the cochlear spiral using a custom ImageJ Measure Line plugin from Eaton-Peabody (https://www.masseyeandear.org/research/otolaryngology/eaton-peabody-laboratories/histology-core). The plugin superimposed frequency correlates on the microdissected spiral image by application of the cochlear frequency map for mice. For cytocochleograms, counting of myosin 7A immunolabeled inner (IHC) and outer (OHC) hair cells was performed in two nearby 200-µm-long segments of the organ of Corti that corresponded to the 5.66, 8.00, 11.32, 16.00, 22.56, 32, and 45.2 kHz regions from *Ocm*^+/+^ and *Ocm*^−/−^ cochleae. Hair cells were considered absent if the stereociliary bundles and cuticular plates were missing, and there was no myosin 7A immunofluorescence. For counts of presynaptic ribbons (CtBP2) and ribbon synapses (paired GluR2 and CtBP2), maximum intensity projections of confocal *z*-stacks either between 16 and 22.6 kHz or between 8 and 12 kHz (apical) of the cochlear spiral was taken. Ribbons were analyzed with the Imaris software (v10.2, Oxford Instruments) using the spots feature to identify puncta and determine their sizes and number. The thresholding criterion for pixel intensity in the CtBP2 channel was set at 30.1 and region threshold set at 191 (for a 8 bit image with a dynamic range of 0–255). The same isointensity criterion was applied to all *z*-stacks in the same projection. A blinded observer counted CtBP2 immunopuncta from the perinuclear region to the synaptic pole in maximum intensity projections. For GCaMP6s and OCM fluorescence intensity, quantification was performed in ImageJ using a ROI from the cytoplasm compared with a similar ROI outside the organ of Corti. After background removal, relative fluorescence intensity was determined. Two to five cochlea from different animals were analyzed per condition.

### Western blotting

Cochleae were dissected and harvested in ice-cold PBS. Cochleae were then transferred into a precooled tube (one cochlea per sample) containing 250 µl ice-cold RIPA-lysis buffer containing as follows: 1% Triton X-100, 25 mM Tris, pH 7.4, 150 mM NaCl, 1 mm DTT, 1 mm MgCl_2_, 1 mM phenylmethylsulfonyl fluoride, and 1× protease inhibitor cocktail (Thermo Fisher Scientific). A homogenizer (Kimble Pellet Pestle, DWK Life Science) was used to crack up cochleae manually, followed by an additional 30 min incubation in lysis buffer. Cochlear lysates were further sonicated (Thermo Fisher Scientific) 3 times × 5 s each on ice. Samples were then transferred to a rotating wheel at 4°C for an additional 60 min; lysates were spun down at 4°C, 12,000 × *g* for 5 min to remove cell debris. The supernatants were then transferred to a new tube and heated for 30 min at 37°C in 4× SDS-sample buffer (Bio-Rad Laboratories, 355 mM β-ME added). Samples were then subject to SDS–PAGE and protein was transferred to a PVDF membrane. Membranes were blocked with 2.5% fish gelatin for 1 h at RT and incubated overnight at 4°C with primary antibody to rabbit P2X2R (1:400, Alomone Labs) and rabbit β-tubulin (1:1,000). After washing, membranes were incubated with appropriate HRP-conjugated secondaries (anti-rabbit 1:7,500) for 1 h at RT. Clarity Max ECL Substrate (Bio-Rad Laboratories) was used to detect chemiluminescence via a ChemiDoc imaging system. Relative protein expression levels (normalized to β-tubulin) were determined by densitometric analysis using the FIJI software.

### Statistical analysis

Statistical analysis was performed using the GraphPad Prism 9 and MATLAB software. Statistical comparisons of means were made by *t* test or, when normal distribution could not be assumed, the Mann–Whitney *U* test. For multiple comparisons, one-way, two-way, and three-way ANOVA was followed by unpaired *t* tests using a pooled variance estimate, with Bonferroni’s correction unless otherwise stated. A significance level of *p* < 0.05 was used to determine statistical significance. Data are given as mean ± SD. Animals of either sex were randomly assigned to the different experimental groups. No statistical methods were used to define the sample size, which was selected based on published similar work. Animals were taken from multiple cages and breeding pairs.

## Results

### OHCs from *Ocm*^−/−^ mice exhibit comparable biophysical profiles and synaptic innervation to *Ocm*^+/+^ mice

**Figure 1.**
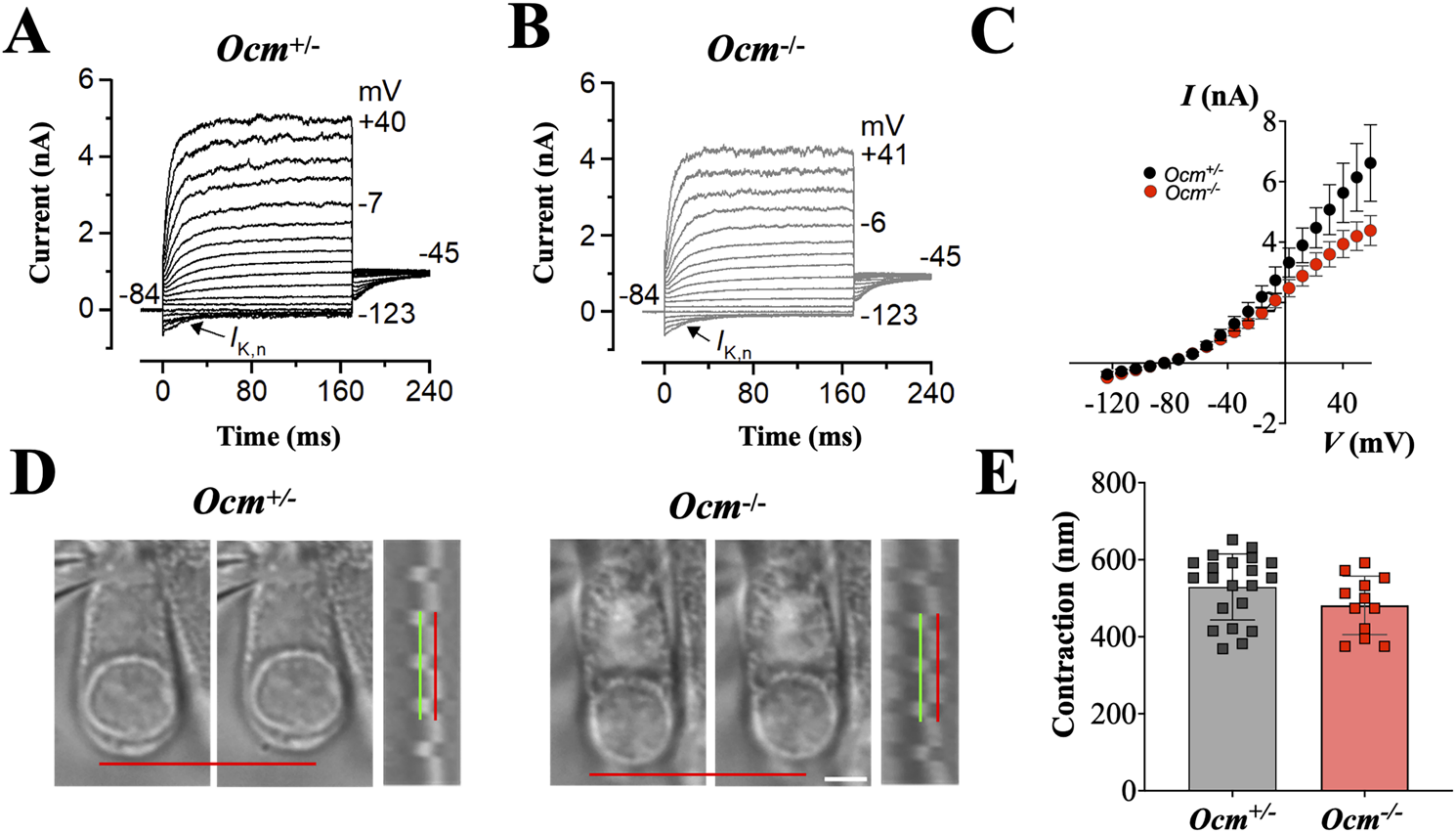
Biophysical properties of OHCs young adult CBA/CaH *Ocm*^−/−^ mice. K^+^ currents from control OHCs (***A***) and *Ocm*^−/−^ (***B***) *Ocm*^+/+^ mice at 1–2 months with a series of 10 mV voltage steps from the holding potential of −84 mV. ***C***, Average current–voltage curves, *n* = 4 for each genotype. ***D***, ***E***, OHC contraction in seven control mice, 21 OHCs, (mean = 529.3 ± 85.53 nm) is shown in open gray squares. OHC contraction in four *Ocm*^−/−^ mice, 12 OHCs (mean = 481.4 ± 75.94 nm), is shown in open red circles. Red and green lines represent OHC length at two membrane potentials, the contraction distance was defined as the difference between these two measurements.

Both Ca_V_1.3 channels and P2X2 receptors play critical roles in shaping the biophysical properties and functional behavior of cochlear hair cells. At prehearing ages, OHCs from *Ocm*^−/−^ mice exhibit normal biophysical profiles (Murtha et al., 2022) but exhibit a downregulation of Ca_V_1.3 channels and upregulation of P2X2 receptors (Yang et al., 2023). Therefore, we investigated whether the lack of OCM had any effect on mature OHC biophysical profiles and electromotility. In 1–2-month-old CBA/CaH mice, we investigated the potassium currents in OHCs while applying a series of hyperpolarizing and depolarizing voltage steps from the holding potential of −84 mV with 10 mV increments (Fig. 1*A*,*B*). The time course and voltage dependence of the outward K^+^ currents in OHCs were mostly comparable between *Ocm*^−/−^ and control OHCs (Fig. 1*A*–*C*). Both genotypes have similar currents at negative voltages suggesting that baseline conductance at hyperpolarized voltages are unchanged by the loss of OCM. However, there was a small, but significant difference (*p* < 0.001, two-wayANOVA) between genotypes at more positive voltages (>−40 mV). Overall, the *I*–*V* curves suggest that the underlying channel type or gating is unchanged but maximum conductance is reduced without OCM. We then tested OHC electromotile responses. Under whole-cell patch–clamp conditions, stepping the membrane potential from −84 to +40 mV caused OHCs to shorten. OHC contraction was not significantly different (*p* = 0.1168, two-way ANOVA) between the two genotypes (Fig. 1*D*,*E*). We found that the lack of OCM minimally affects OHC electromotility in young adult mice.

**Figure 2.**
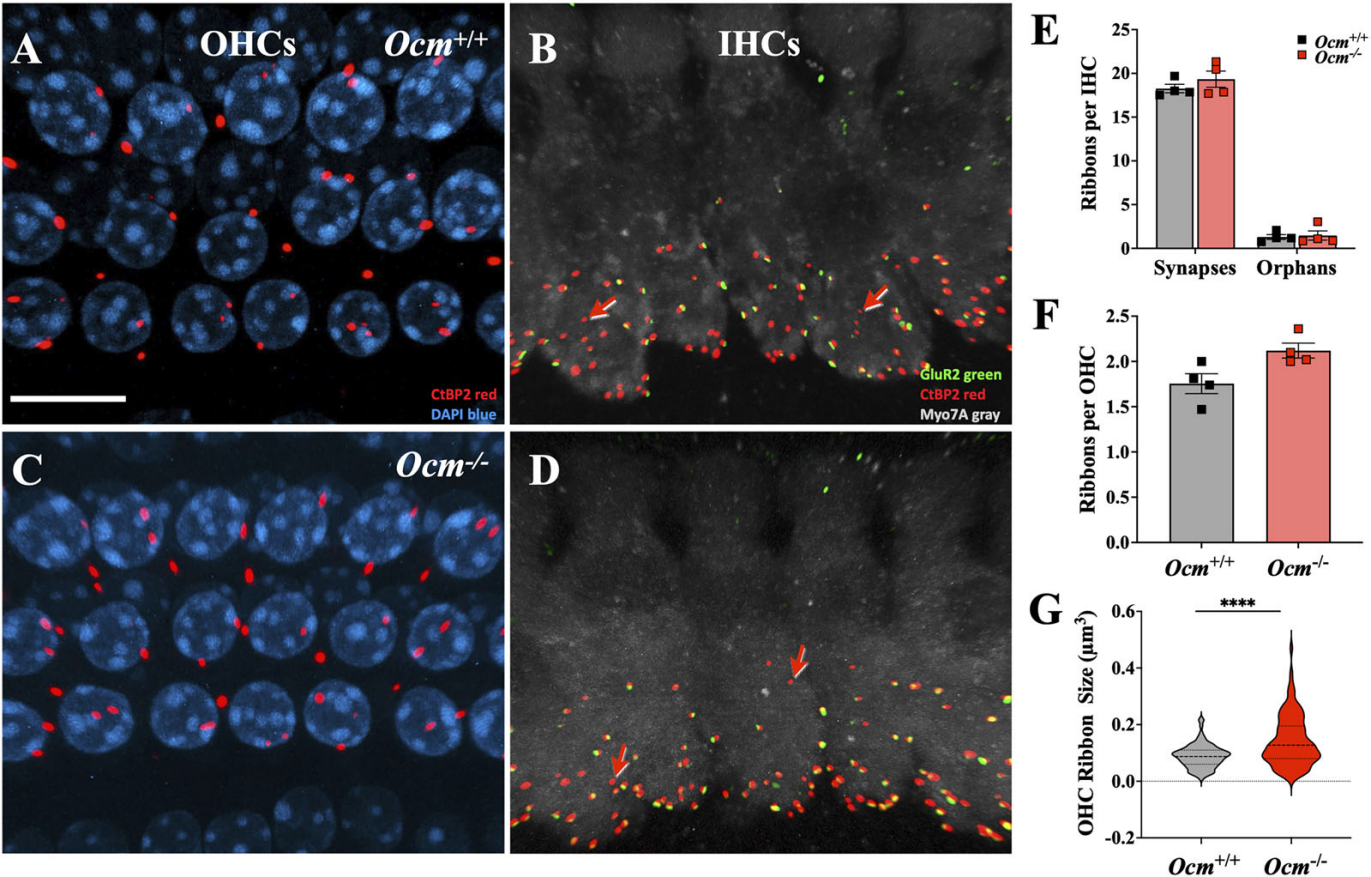
Normal abundance but altered size of OHC ribbons in adult *Ocm*^−/−^ mice. Maximum projections of confocal *z*-stacks from the 16 kHz region in 1-month-old *Ocm^+/+^* (***A***, ***B***) and *Ocm^−/−^* (***C***, ***D***) cochlea. Cochlear whole mounts were immunolabeled for presynaptic ribbons (CtBP2, red), postsynaptic glutamate receptors (GluR2; green), hair cells (myosin 7A; gray), and nuclei (Hoechst, blue). ***A***, ***C***, Presynaptic ribbons in the OHC region in *Ocm^+/+^* and *Ocm^−/−^* mice. ***B***, ***D***, Juxtaposed CtBP2 and GluR2 puncta in IHCs as well as orphan ribbons (CtBP2 only, red arrows). ***E***, IHC synapse counts in *Ocm^+/+^* and *Ocm^−/−^* mice. ***F***, OHC ribbon counts in *Ocm^+/+^* and *Ocm^−/−^* mice. ***G***, OHC ribbon volumes in *Ocm^+/+^* and *Ocm^−/−^* mice.

Hair cells have specialized presynaptic ribbons that facilitate neurotransmitter release. In the mature mouse cochlea, each OHC is innervated by two to three type II afferent fibers associated with presynaptic ribbons, while there are 10–20 type I fibers contacting each IHC in adult mice (Huang et al., 2012; Ceriani et al., 2019; Johnson et al., 2019). During the prehearing period, apical coil OHCs from *Ocm*^−/−^ mice show increased numbers of ribbons and type II afferent contacts (Yang et al., 2023). We investigated whether synapses and ribbons were altered in IHCs and OHCs in the *Ocm*^−/−^ young adult mice. Using CtBP2 to label presynaptic ribbons and GluR2 antibodies to label the closely apposed postsynaptic receptor patches (present only in type I terminals), we focused on midcochlear (16–22.6 kHz) regions (Fig. 2*A*–*D*). Although the number of ribbons and receptor patches trended higher in *Ocm*^−/−^ mice, they were not statistically different. In IHCs, the mean number of ribbons was 19.3 ± 1.8 in *Ocm*^−/−^ mice compared with 18.3 ± 1.0 (*p* = 0.4058) in *Ocm*^+/+^ mice (Fig. 2*E*). There was also no statistical difference in the mean number of orphan ribbons in *Ocm*^−/−^ IHCs (1.5 ± 1.1) compared with *Ocm*^+/+^ IHCs (1.3 ± 0.6; *p* = 0.974). The mean number of OHC ribbons was 2.3 ± 0.2 in *Ocm*^−/−^ mice compared with 2.0 ± 0.3 (*p* = 0.114) in *Ocm*^+/+^ mice (Fig. 2*F*). However, ribbon volumes were larger in *Ocm*^−/−^ OHCs (0.14 ± 0.09 µm^3^) compared with *Ocm*^+/+^ OHCs (0.09 ± 0.04 µm^3^; *p* < 0.0001; two-way ANOVA; Fig. 2*G*). Both IHC synapses and OHC ribbons are mostly unchanged in the absence OCM, suggesting there should be minimal changes in afferent neural responses.

### Lack of OCM alters temporal coding and increases PTS after noise exposure

**Figure 3.**
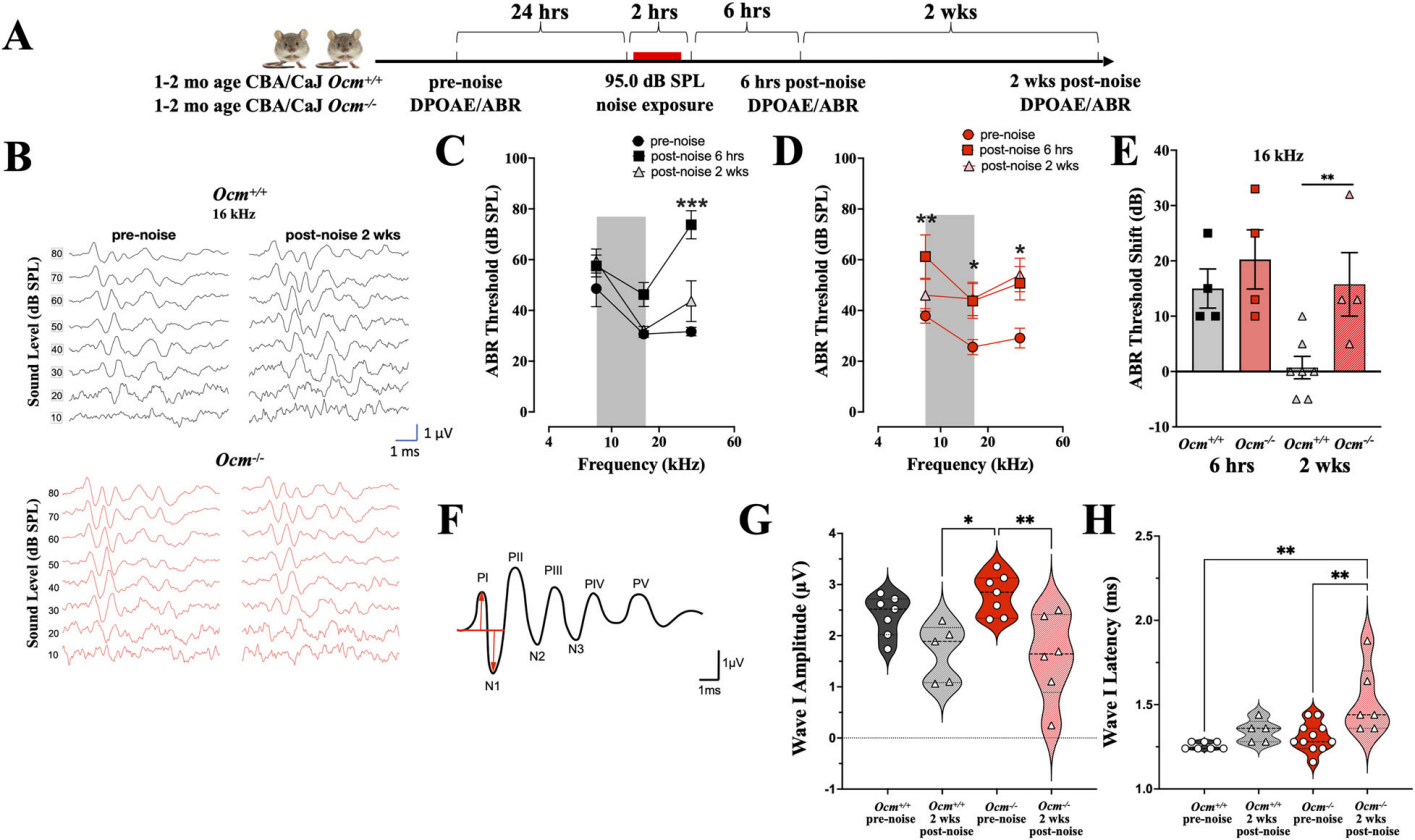
*Ocm^−/−^* mice showed increased ABR threshold shifts after noise. ***A***, Schematic of experimental timeline. ***B***, Representative ABR waveforms at 16 kHz from *Ocm^+/+^* and *Ocm^−/−^* mice before and 2 weeks after noise. ***C***, ***D***, Mean ABR thresholds (±SD) from *Ocm^+/+^* (***C***) and *Ocm^−/−^* (***D***) mice at 8, 16, and 32 kHz before, 6 h, and 2 weeks after noise exposure. Gray shading shows the exposure band. Group sizes were four for each genotype. Asterisks (*) indicate significant differences between pre- and 6 h postnoise, hash marks (#) indicate significant differences between pre- and 2 weeks postnoise. ***E***, Mean ABR threshold shifts (±SD) at 16 kHz, 6 h, and 2 weeks after noise exposure. ***F***, An idealized ABR waveform showing positive and negative peaks. ***G***, ***H***, Violin plots of amplitude (P1–N1) and latency of ABR P1 evoked by a 16 kHz tone pip at 80 dB SPL. Significance values are coded as follows: ^*/#^*p* < 0.05; ^**/##^*p* < 0.01; ^***/###^*p* < 0.001.

We investigated whether the absence of OCM in OHCs increases vulnerability to moderate levels of noise exposure in young adult mice. We used an octave-band noise (8–16 kHz) at 95 dB SPL for 2 h, designed to cause a large temporary threshold shift (TTS) with minimal or no PTS (Hirose and Liberman, 2003; Fernandez et al., 2010; Fig. 3*A*). To assess cochlear function, we measured ABRs and DPOAE in anesthetized mice. Since genetic disruption of *Ocm* leads to progressive hearing loss (Climer et al., 2021), noise exposure was performed in mice that were 4–9 weeks, which is well before the appearance of ABR threshold elevation. We compared ABR amplitudes, latencies, and thresholds before and again at 6 h and 2 weeks after noise exposure. Prior to noise exposure, ABR thresholds were similar between *Ocm^+/+^* and *Ocm*^−/−^ mice (Fig. 3*B*–*D*). At 6 h postnoise exposure, ABR thresholds at 32 kHz showed a significant increase in *Ocm*^+/+^ mice (*p* < 0.001, two-way ANOVA; Fig. 3*C*), while in *Ocm*^−/−^ mice thresholds at all three test frequencies were significantly elevated (*p* = 0.0098 for 8 kHz; *p* = 0.0416 for 16 and *p* = 0.0164 for 32 kHz, two-way ANOVA; Fig. 3*D*). ABR threshold shifts at 16 kHz were comparable between *Ocm*^+/+^ and *Ocm*^−/−^ mice 6 h after noise exposure. However, after 2 weeks, thresholds in *Ocm*^−/−^ mice did not recover at 16 kHz in contrast to wild types (*p* = 0.0135; Fig. 3*E*).

Prior to noise exposure, *Ocm*^−/−^ mice showed larger variability in the amplitude of ABR wave I (N1–P1 in Fig. 3*F*) than *Ocm^+/+^* mice. Focusing on 16 kHz at 80 dB SPL, coefficients of variation (CVs) for wave I amplitudes were 53% in *Ocm*^−/−^ mice compared with 17% in wild types. As shown in Figure 3*G*, there was a bimodal distribution of wave I amplitudes in *Ocm*^−/−^ mice prior to noise exposure, with one peak at 2.8 µV (±0.40; *N* = 7; CV = 14%) and a second at 0.75 µV (±0.12; *N* = 4; CV = 16%). *Ocm*^−/−^ mice had a mean wave I amplitude of 2.1 ± 1.082 µV versus 2.4± 0.40 µV for *Ocm*^+/+^ mice. In contrast, before exposure, *Ocm*^−/−^ and *Ocm*^+/+^ mice showed similar latencies for wave I (1.3 ± 0.09 ms and 1.3 ± 0.02 ms, respectively; Fig. 3*H*).

Two weeks after exposure, *Ocm*^−/−^ and *Ocm*^+/+^ mice showed greater variation in ABR responses (Fig. 3*G*,*H*). The largest differences between mutant and wild-type mice were seen in their wave I amplitudes. In both *Ocm*^−/−^ and *Ocm*^+/+^ mice, wave I amplitudes decreased (*Ocm*^−/−^ 1.6 ± 0.84 µV vs *Ocm*^+/+^ 1.7 ± 0.56 µV) but only significantly so from prenoise values in the *Ocm^+/+^* ears (*p* = 0.0480; Fig. 3*G*). Again, *Ocm*^−/−^ mice demonstrated higher CV (53%) compared with *Ocm*^+/+^ mice (33%). As shown in Figure 3*H*, after the noise, *Ocm*^−/−^ mice demonstrated a significant increase in mean latency for wave I (Δ = 1.5 ± 0.20 ms; *p* < 0.0001, two-way ANOVA). *Ocm*^+/+^ mice had a smaller but statistically significant increase in wave I latency (Δ = 1.34 ± 0.067 ms; *p* = 0.0189).

**Figure 4.**
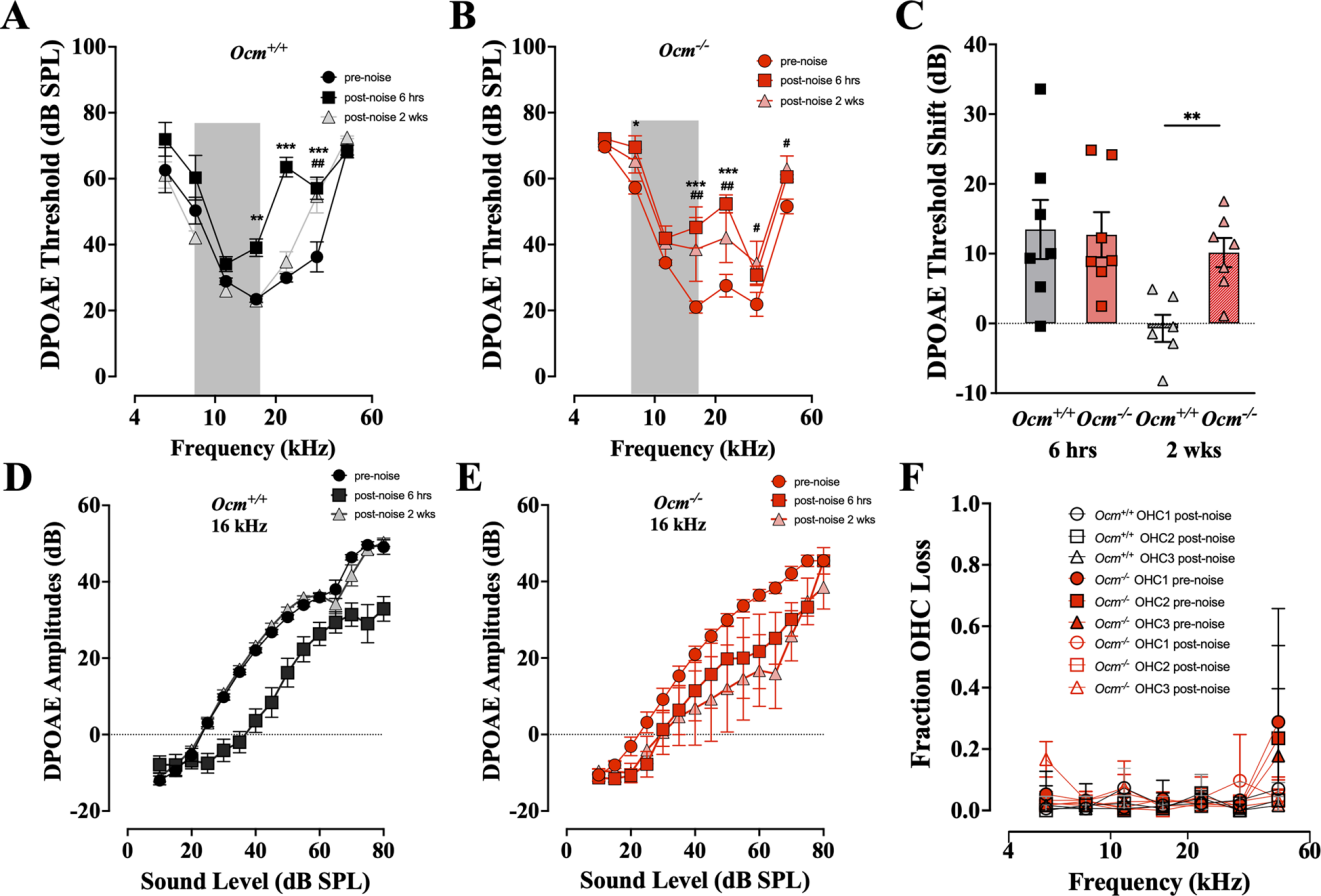
*Ocm^−/−^* mice showed increased DPOAE threshold shifts after noise. ***A***, ***B***, Mean DPOAE thresholds (±SD) before, 6 h after, and 2 weeks after noise. *n* = 11 for *Ocm^+/+^*; *n* = 7 for *Ocm^−/−^*. Significance values displayed as described for Figure 3. ***C***, DPOAE threshold shifts averaged across all test frequencies, showing means (±SD) and individual animals. ***D***, ***E***, Mean DPOAE amplitude versus level functions (±SEM). ***F***, Mean fractional OHC losses (±SD) in different experimental groups and OHC rows as indicated in the key.

DPOAE thresholds were also significantly increased in both *Ocm*^+/+^ and *Ocm*^−/−^ mice 6 h after noise exposure (*p* < 0.0001 for *Ocm*^+/+^ and *Ocm*^−/−^ mice, two-way ANOVA; Fig. 4*A*). After 2 weeks, DPOAE thresholds from *Ocm*^+/+^ mice had recovered to prenoise thresholds, except at 32 kHz (*p* < 0.0001, two-way ANOVA). In contrast, *Ocm*^−/−^ mice still exhibited elevated DPOAE thresholds at high frequencies (16 kHz, *p* = 0.0015; 22 kHz, *p* = 0.0031; 32 kHz, *p* *=* 0.0159; 45 kHz, *p* = 0.0202; Fig. 4*B*). The mean DPOAE threshold shifts across all frequencies, 6 h after noise were comparable between *Ocm*^+/+^ and *Ocm*^−/−^ mice (Fig. 4*C*). Two weeks after noise, *Ocm*^−/−^ mice showed significantly larger DPOAE threshold shifts than *Ocm*^+/+^ mice (*p* = 0.0172, two-way ANOVA; Fig. 4*C*). DPOAE suprathreshold amplitudes at 16 kHz were reduced in both *Ocm*^+/+^ and *Ocm*^−/−^ mice at 6 h postnoise (Fig. 4*D*,*E*). However, 2 weeks after noise, *Ocm*^−/−^ mice showed reduced suprathreshold DPOAE amplitudes, while *Ocm*^+/+^ mice recovered. The near complete recovery from exposure in wild types suggests that OCM may be involved in protection from PTS after moderate noise exposures.

PTS after noise are often associated with damaged or missing OHCs, whereas TTS is not (Liberman and Mulroy, 1982). We assessed OHC loss 2 weeks after noise exposure. As expected, 95 dB SPL noise produced no significant OHC loss in *Ocm*^+/+^ mice (Fig. 4*F*). Similarly, the *Ocm*^−/−^ mice demonstrated no evidence of OHC loss in either control or exposed ears except at 45 kHz, the highest frequency evaluated. Although *Ocm*^−/−^ mice showed significant ABR (Fig. 3*E*) and DPOAE (Fig. 4*C*) threshold shifts across frequency regions at 2 weeks postexposure, there was little hair cell loss associated with this PTS except in the 45 kHz region (Fig. 4*F*).

### Lack of OCM increases threshold shifts following prolonged noise exposure

**Figure 5.**
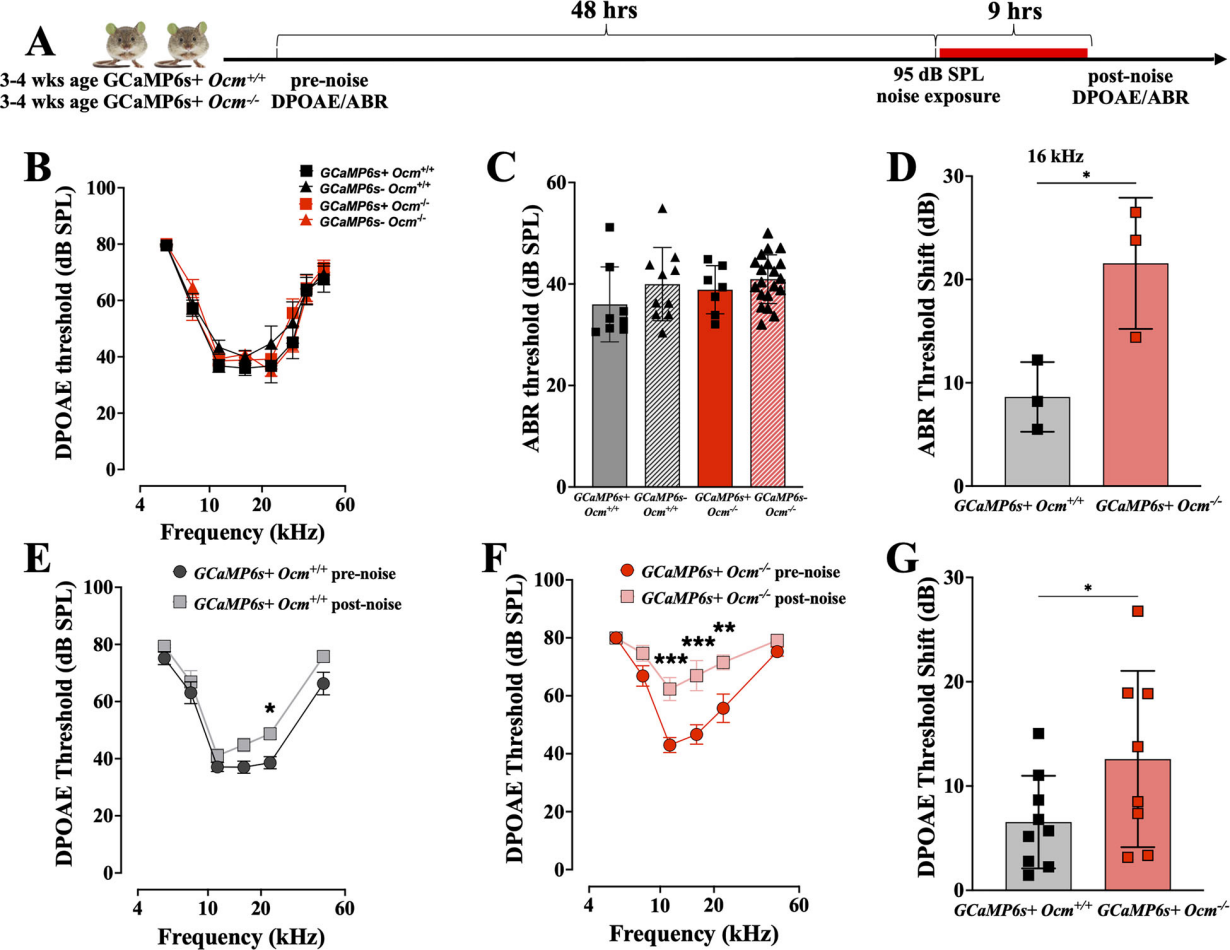
*Ocm^−/−^* mice showed increased susceptibility after prolonged noise exposure. ***A***, Schematic of the timeline for Ca^++^ imaging experiments. ***B***, Mean DPOAE thresholds (±SD) from group sizes of 10 *Ocm*^+/+^ GCaMP6s^−^, 8 *Ocm*^+/+^ GCaMP6s^+^, 20 *Ocm^−/−^* GCaMP6s^−^, and 7 *Ocm^−/−^* GCaMP6s^+^ mice. ***C***, Mean ABR thresholds (±SD) at 16 kHz. ***D***, Mean ABR threshold shift (±SD) at 16 kHz before and after noise exposure. ***E***, ***F***, Mean DPOAE thresholds (±SD) before and after noise exposure. *n* = 6 for each genotype. ***G***, Mean DPOAE threshold shifts (±SD) averaged across all frequencies. Significance in all panels shown as asterisks, *p < 0.05; **p < 0.01; ***p < 0.001.

Noise exposure dramatically increases the levels of intracellular free Ca^2+^ in OHCs (Fridberger et al., 1998; Jacob et al., 2013). Additionally, studies show that 6–24 h of continuous moderate noise induces a stable, protective cochlear adaptation dominated by sustained purinergic signaling and gain suppression (Wang et al., 2003). To investigate Ca^2+^ dynamics and purinergic receptor expression, we expressed a tissue-specific Ca^2+^ sensor (Atoh1-GCaMP6s) in Ocm^+/+^ and Ocm^−/−^ mice. Since the loss of OCM also causes increased levels of intracellular free Ca^2+^ in postnatal mice (Murtha et al., 2022), we hypothesized that the early progressive hearing loss observed in adult *Ocm*^−/−^ mice is partly due to sustained Ca^2+^ overload in OHCs. We compared GCaMP6s + *Ocm^+/+^* and *Ocm^−/−^* in their susceptibility with prolonged (9 h) broadband noise exposure at 95 dB SPL. Cochlear thresholds were measured 1 h following noise exposure (Fig. 5*A*). As a Ca^2+^ sensor, GCaMP6s contains a calmodulin domain that might also disrupt calcium homeostasis over time. However, at 3–4 weeks of age, neither the endogenous expression of GCaMP6s nor the absence of OCM affects DPOAE thresholds at any frequency [*p* = 0.6928 for *Ocm*^+/+^ vs *Ocm*^−/−^; *p* = 0.2887 for GCaMP6s(−) vs GCaMP6s(+), three-way ANOVA; Fig. 5*B*]. We measured ABR thresholds only at 16 kHz, and before exposure there were no significant differences among GCaMP6s(−) and GCaMP6s(+), *Ocm^+/+^* and *Ocm^−/−^* mice (*p* = 0.4744, two-way ANOVA; Fig. 5*C*). After 9 h of exposure at 95 dB SPL, GCaMP6s + *Ocm^−/−^* mice showed a larger ABR threshold shift (*p* = 0.0178, *t* test; Fig. 5*D*). In GCaMP6s + *Ocm^+/+^* mice, DPOAE thresholds were significantly elevated only at 22 kHz (*p* = 0.0430; two-way ANOVA; Fig. 5*E*). However, in GCaMP6s + *Ocm^−/−^* mice, DPOAE thresholds were elevated at 11 (*p* = 0.0010), 16 (*p* < 0.0010), and 22 kHz frequencies (*p* = 0.0027; Fig. 5*F*). The mean DPOAE threshold shift across all frequencies was significantly higher in GCaMP6s + *Ocm^−/−^* mice compared with GCaMP6s + *Ocm^+/+^* (*p* = 0.04, *t* test; Fig. 5*G*). Thus, GCaMP6s + *Ocm*^−/−^ mice demonstrated similar vulnerability to prolonged broadband noise as CBA/CaJ *Ocm*^−/−^ mice did for shorter duration octave-band noise exposure.

### Prolonged noise exposure induces higher GCaMP6s signaling in *Ocm*^−/−^ mice

**Figure 6.**
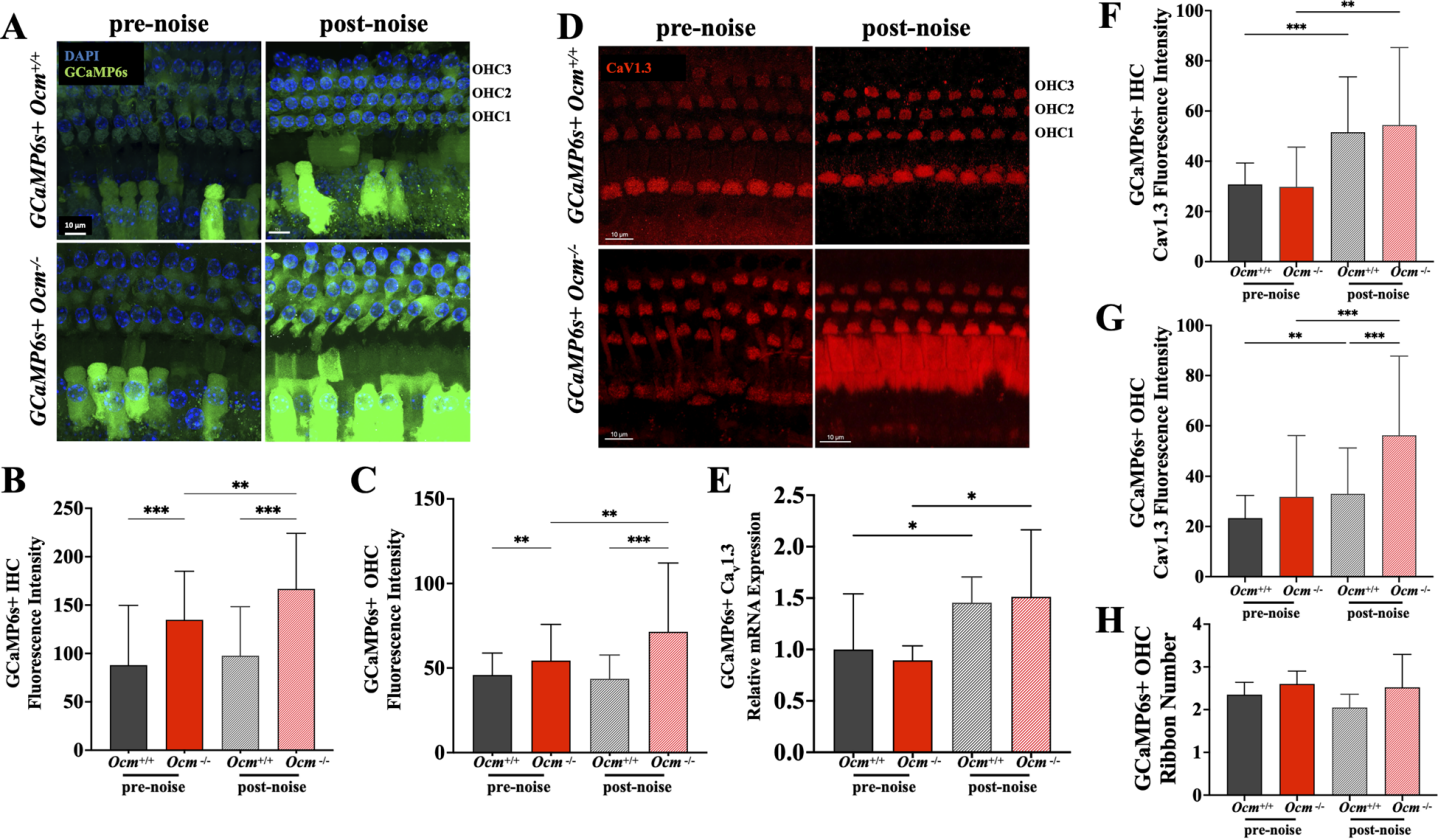
Prolonged noise exposure alters GCaMP6s signaling and Ca_V_1.3 expression. ***A***, Maximum projections of the Ca^++^ signal in confocal *z*-stacks from GCaMP6s + *Ocm*^+/+^ and *Ocm*^−/−^ apical turns before and after prolonged noise exposure. ***B***, ***C***, Relative fluorescence intensity in IHCs and OHCs, respectively, before and after noise. ***D***, Maximum projection of Ca_V_1.3 immunolabeling in *z*-stacks from the apical turn in GCaMP6s^+^ *Ocm*^+/+^ and *Ocm*^−/−^ before and after prolonged noise exposure. ***E***, qRT-PCR shows that Ca_V_1.3 expression is comparable between GCaMP6s + *Ocm^+/+^* and *Ocm^−/−^* mice and that prolonged noise significantly increased Ca_V_1.3 expression in both GCaMP6s + *Ocm^+/+^* and *Ocm^−/−^* cochleae. Three animals for each genotype under each condition. Mean values (±SD) were normalized to GCaMP6s + *Ocm^+/+^* prenoise. ***F***, ***G***, Mean (±SD) relative fluorescence for IHCs and OHCs, respectively. ***H***, Mean ribbon counts (±SD) for OHCs in *Ocm^+/+^* and *Ocm^−/−^* mice. Significance indicated as follows: *p < 0.05; **p < 0.01; ***p < 0.001, one-way ANOVA followed by Kruskal–Wallis test.

Both GCaMP6s *+* *Ocm*^+/+^ and *Ocm^−/−^* mice showed endogenous GCaMP6s fluorescence in the cochlea under confocal microscopy (Fig. 6*A*). As shown in Figure 6*B*, GCaMP6s + *Ocm^−/−^* IHCs exhibited higher fluorescence both before and after noise compared with GCaMP6s + *Ocm*^+/+^ IHCs. Before noise exposure, IHCs from GCaMP6s + *Ocm*^+/+^ and *Ocm^−/−^* mice had relative fluorescent intensities of 88.0 ± 61.74 and 134.8 ± 50.09 (*p* = 0.0003), respectively. After noise, there was a modest increase (10.96%) in fluorescent intensities in GCaMP6s + *Ocm*^+/+^ IHCs (97.7 ± 50.65; *p* = 0.8339, Kruskal–Wallis test). This contrasted with IHCs from GCaMP6s + *Ocm^−/−^* mice, which showed a 23.66% increase after noise (166.7 ± 57.51; *p* = 0.0276, Kruskal–Wallis test). Similarly before noise exposure, GCaMP6s + *Ocm^−/−^* OHCs had significantly higher baseline fluorescence intensity than GCaMP6s + *Ocm*^+/+^ OHCs. As shown in Figure 6*C*, relative fluorescence intensity for OHCs in GCaMP6s + *Ocm*^+/+^ mice was 45.9 ± 12.98, while in GCaMP6s + *Ocm^−/−^* mice, it was 54.4 ± 21.38 (*p* = 0.0280, Kruskal–Wallis posttest). However, immediately following noise exposure, there was a 31.18% increase in GCaMP6s + *Ocm*^−/−^ OHCs fluorescence (71.4 ± 40.70; *p* = 0.0060, Kruskal–Wallis posttest), while little or no change (−4.9%) in GCaMP6s + *Ocm*^+/+^ OHCs (43.6 ± 14.07; *p* > 0.9990, Kruskal–Wallis posttest). The increased fluorescence following noise in GCaMP6s + *Ocm^−/−^* OHCs (Fig. 6*C*) may indicate a higher basal level of intracellular Ca^2+^ signaling due to the lack of OCM (Murtha et al., 2022; Yang et al., 2023). Altogether, the lack of OCM in OHCs led to higher Ca^2+^ signaling in both IHCs and OHCs prior to and following prolonged, moderate noise exposures.

### Changes in Ca_V_1.3 and synaptic ribbons after prolonged noise exposure

The increased fluorescence intensities observed in GCaMP6s + IHCs and OHCs could be due to altered contributions from voltage-gated Ca^2+^ channels. In the *Ocm*^−/−^ cochlea during development, previous studies report a downregulation of Ca_V_1.3 (Yang et al., 2023), the predominant voltage-gated Ca^2+^ channel in hair cells (Michna et al., 2003; Hafidi and Dulon, 2004). We investigated whether the lack of OCM and noise exposure altered their expression in the adult cochlea (Fig. 6*D*). qRT-PCR showed no difference in relative Ca_V_1.3 mRNA expression (*CACNA1D*) between GCaMP6s + *Ocm*^+/+^ and *Ocm*^−/−^ cochleae (Fig. 6*E*; *n* = 3 for each genotype; *p* = 0.1118, Kruskal–Wallis test). Prolonged noise increased *Ca_V_1.3* mRNA expression in both GCaMP6s + *Ocm*^+/+^ and *Ocm*^−/−^ cochleae (Fig. 6*E*; *n* = 3 for each genotype; *p* = 0.0189 for GCaMP6s + *Ocm*^+/+^; *p* = 0.0146 for GCaMP6s + *Ocm*^−/−^, Kruskal–Wallis test). There was no significant difference between GCaMP6s + *Ocm*^+/+^ and *Ocm*^−/−^ cochleae postnoise (*p* = 0.2492, Kruskal–Wallis test). Unlike endogenous GCaMP6s fluorescence, Ca_V_1.3 immunofluorescence microscopy showed that there was no significant difference in IHCs or OHCs from GCaMP6s + *Ocm*^+/+^ and *Ocm*^−/−^ mice (Fig. 6*F*,*G*; for prenoise *Ocm*^+/+^, 23.33 ± 9.063; *n* = 94; for prenoise *Ocm*^−/−^: 31.85 ± 24.39; *n* = 79; *p* = 0.6545, Kruskal–Wallis test). Prolonged noise increased Ca_V_1.3 fluorescence in both GCaMP6s + *Ocm*^+/+^ and *Ocm*^−/−^ IHCs (Fig. 6*F*, for prenoise *Ocm*^+/+^, 30.75 ± 8.517; *n* = 40; for prenoise *Ocm*^−/−^, 29.79 ± 15.83; *n* = 24; *p* > 0.9999, Kruskal–Wallis test). Both GCaMP6s + *Ocm*^+/+^ and *Ocm*^−/−^ IHCs exhibited higher fluorescence after prolonged noise exposure (Fig. 6*F*, for postnoise *Ocm*^+/+^, 51.55 ± 22.06; *n* = 36; *p* = 0.0001; for postnoise *Ocm*^/−^, 54.42 ± 30.87; *n* = 39; *p* = 0.0042; Kruskal–Wallis test). Similar to IHCs, there was no significant difference between prenoise GCaMP6s + *Ocm*^+/+^ and *Ocm*^−/−^ OHCs (Fig. 6*G*; for postnoise *Ocm*^+/+^, 33.04 ± 18.26; *n* = 111; *p* = 0.0053; for *Ocm*^−/−^, 56.36 ± 31.40; *n* = 96; *p* < 0.0001, Kruskal–Wallis test). Altogether, the lack of OCM had little to no effect on pre- and postnoise levels of Ca_V_1.3 expression.

When evaluated shortly after noise exposure, there can be an increase in the number of presynaptic ribbons in OHCs (Wood et al., 2021). We also investigated the response of OHC ribbons immediately following prolonged noise exposure in apical regions of the cochlea (Fig. 6*H*). We found no significant effects of noise exposure or genotype. Before exposure, the average number of CtBP2 puncta per OHC was 2.4 ± 0.29 in *Ocm*^+/+^ versus 2.6 ± 0.30 in *Ocm*^−/−^ mice. Immediately following exposure, ribbon counts were 2.1 ± 0.31 in *Ocm*^+/+^ and 2.52 ± 0.77 (*p* > 0.9999) in *Ocm*^−/−^ mice. Similar to short-duration exposures, there was no statistical significance between the number of ribbons in *Ocm*^+/+^ or *Ocm*^−/−^ OHCs.

### ATP-induced Ca^2+^ transients in mature OHCs are higher in *Ocm*^−/−^ mice and after prolonged noise exposure

**Figure 7.**
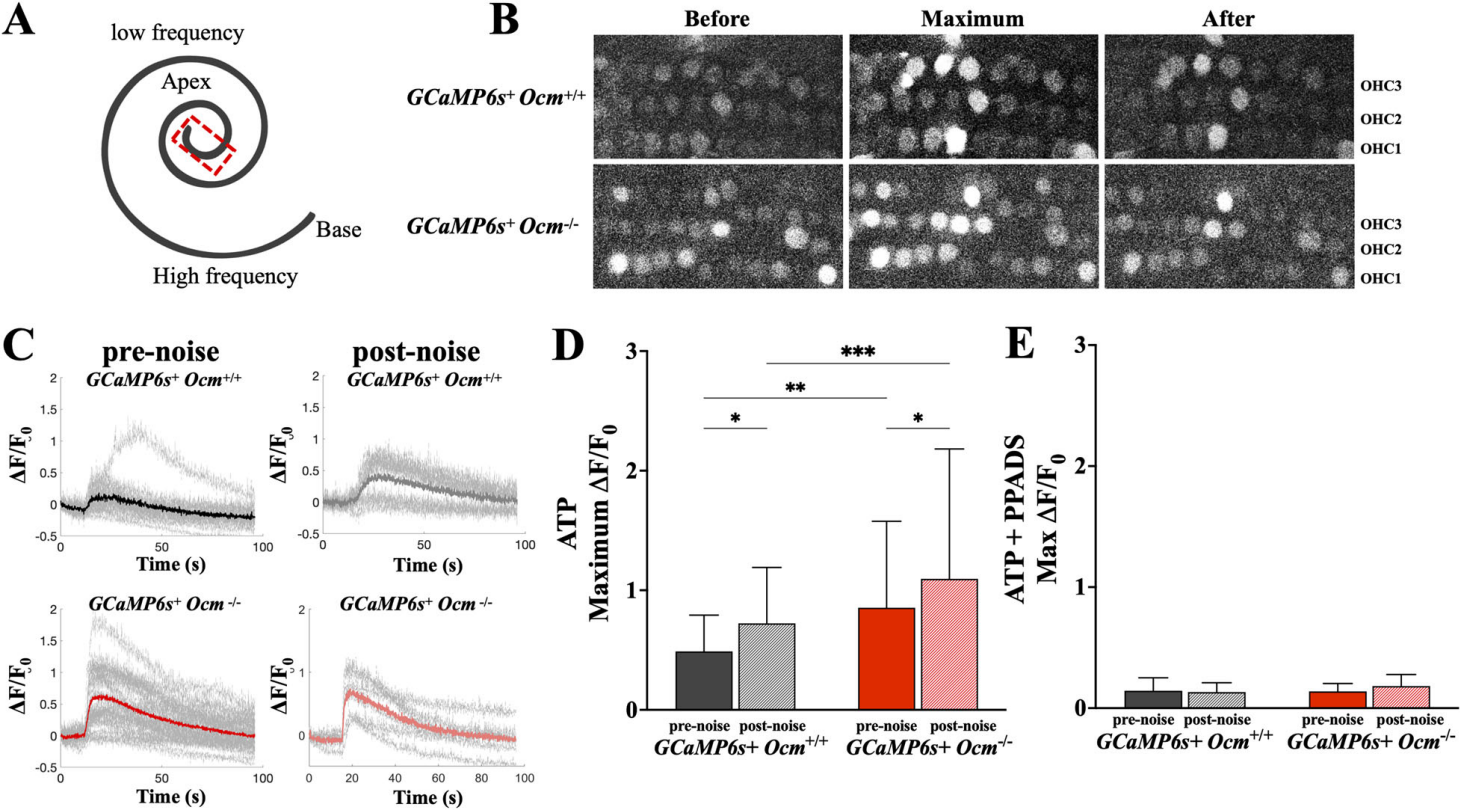
The absence of OCM alters ATP-induced Ca^2+^ transient in OHCs similar to noise. ***A***, Apical turns taken from GCaMP6s + mice at 3–4 weeks for Ca^2+^ transient imaging. ***B***, Representative images from prenoise *Ocm^+/+^* and *Ocm^−/−^* mice showing Ca^2+^ in OHCs before, during, and after 100 μM extracellular ATP. ***C***, Representative traces of fluorescence fractional change (Δ*F*/*F*_0_) in *Ocm^+/+^* and *Ocm^−/−^* OHCs pre- or postnoise exposure (light gray lines) and the mean values (bold lines). ***D***, ***E***, Maximum Δ*F*/*F*_0_ in OHCs from *Ocm^+/+^* and *Ocm^−/−^* mice pre- or postnoise exposure induced by 100 μM ATP or with the presence of PPADS (100 μM). In ***D***, *n* = 4 mice for each genotype under each treatment, with 73 and 93 OHCs from prenoise *Ocm^+/+^* and *Ocm^−/−^* mice, and 83 and 49 OHCs from postnoise *Ocm^+/+^* and *Ocm^−/−^* mice, respectively. For ***E***, *n* = 2 mice for each genotype under each treatment, with 22 and 29 OHCs from prenoise *Ocm^+/+^* and *Ocm^−/−^*, and 15 and 13 OHCs from postnoise *Ocm^+/+^* and *Ocm^−/−^* mice. *p < 0.05; **p < 0.01; ***p < 0.001.

To assess Ca^2+^ dynamics in *Ocm^+/+^* and *Ocm^−/−^* mice with GCaMP6s expression, apical cochleae from 3–4-week-old mice were dissected (Fig. 7*A*) and immediately transferred to the microscope chamber. ATP (100 μM) was extracellularly applied to induce Ca^2+^ transients (Fig. 7*B*), which were measured as fractional changes of GCaMP6s fluorescence (Δ*F*/*F*_0_; Fig. 7*C*, gray line). OHCs from *Ocm^−/−^* mice showed significantly larger ATP-induced Ca^2+^ transients compared with *Ocm^+/+^* littermate (*p* = 0.0015, post hoc test from two-way ANOVA; Fig. 7*D*).

Furthermore, we investigated whether the higher susceptibility of *Ocm^−/−^* mice to the prolonged (9 h) noise exposure was a result of increased Ca^2+^ signaling in OHCs (Murtha et al., 2022; Yang et al., 2023). We found prolonged noise stimulation caused a further increase in maximum Δ*F*/*F*_0_ in OHCs from both genotypes (*p* = 0.0030, two-way ANOVA; Fig. 7*D*). However, OHCs from *Ocm^−/−^* mice exhibited a higher maximum Δ*F*/*F*_0_ signal after noise compared with *Ocm^+/+^* mice (*p* = 0.0008, post hoc test from two-way ANOVA; Fig. 7*D*). The maximum Δ*F*/*F*_0_ signals between prenoise OHCs in *Ocm^−/−^* mice and postnoise OHCs in *Ocm^+/+^* mice were similar (*p* = 0.0015, post hoc test from two-way ANOVA; Fig. 7*D*).

To test whether the ATP and noise-induced Ca^2+^ responses in OHCs were mediated by purinergic receptors, we used the nonselective purinergic receptor antagonist PPADS (100 μM). Extracellular application of PPADS nearly abolished ATP-induced Ca^2+^ transients in OHCs and eliminated the noise-induced difference in the maximum ΔF/F_0_ between *Ocm^+/+^* and *Ocm^−/−^* (*p* = 0.2703, two-way ANOVA; Fig. 7*E*). These data indicate that the lack of OCM increases ATP-induced Ca^2+^ transients in mature OHCs, which mimic the effects caused by long noise exposure.

### Lack of OCM and prolonged noise stimulation upregulate P2X2 receptors

**Figure 8.**
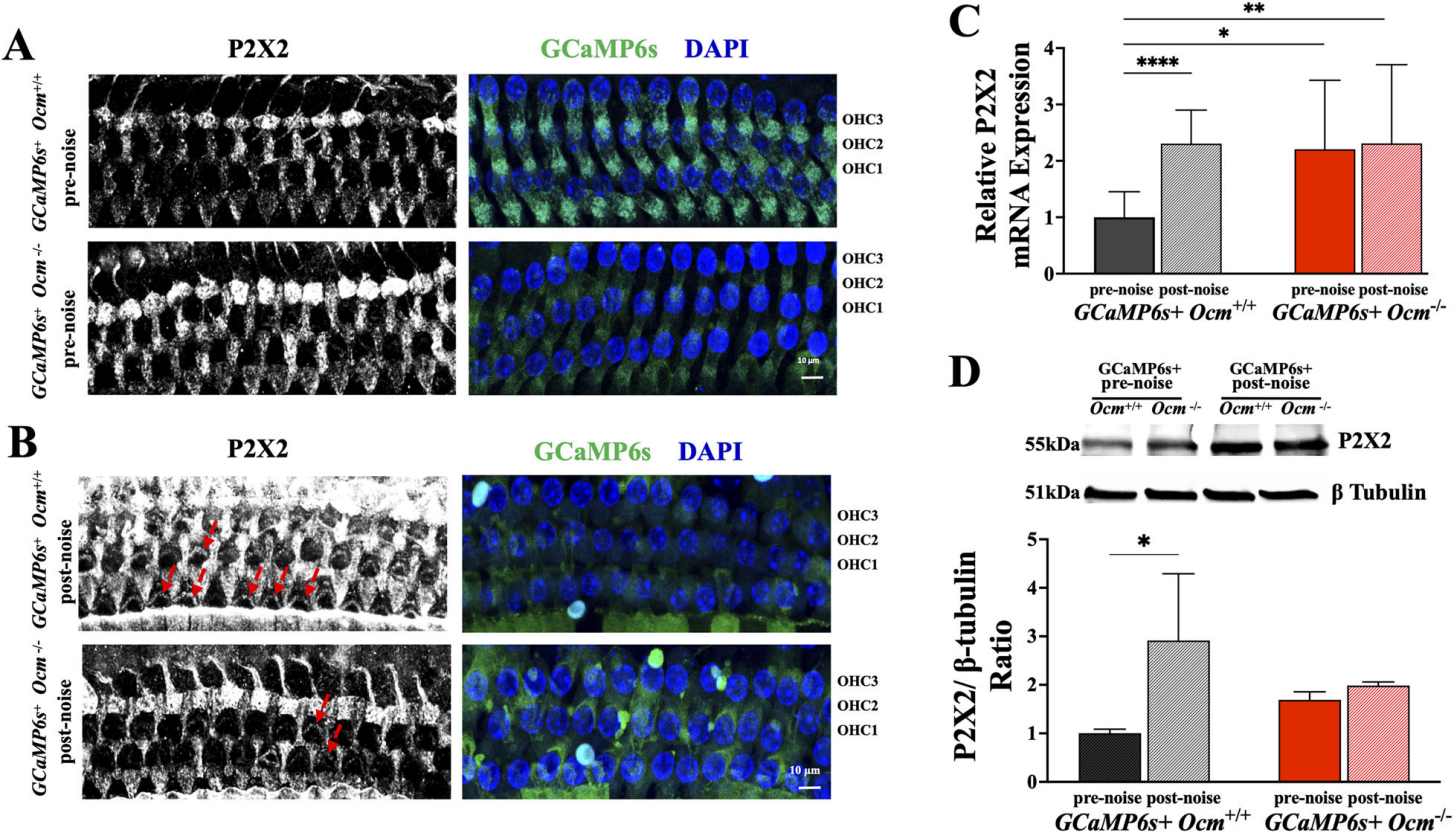
P2X2 expression was upregulated in *Ocm^+/+^* but not *Ocm^−/−^* mice after noise. ***A***, ***B***, Maximum projections of P2X2 immunolabeling in OHCs harvested from pre- and postnoise *Ocm^+/+^* and *Ocm^−/−^* mice. P2X2 (white), GCaMP6s (green), and DAPI (blue) are shown. Red arrows point to the OHC stereocilia. ***C***, Mean P2RX2 expression (±SD) in *Ocm^+/+^* and *Ocm^−/−^* cochleae at 3–4 weeks with or without noise exposure, normalized to prenoise *Ocm^+/+^*. *n* = 3 for each genotype under each experimental condition. ***D***, Western blots of P2X2 protein levels in individual cochlea from pre- or postnoise *Ocm^+/+^* and *Ocm^−/−^* mice at 3–4 weeks. *n* = 3 for each genotype under each experiment condition; β-tubulin (loading control) was used for normalization; all values were then normalized to prenoise *Ocm^+/+^*. Significance values are shown as follows: *p < 0.05; **p < 0.01.

In mature hair cells, several studies report that sustained noise exposure increases levels of purinergic receptor expression, specifically, *P2RX2* levels in the organ of Corti (Wang et al., 2003; Telang et al., 2010). During development, *Ocm*^−/−^ mice also have increased expression of P2X2 receptors at least through the onset of hearing (Yang et al., 2023). We tested whether prolonged noise would further increase purinergic expression, leading to larger Ca^2+^ responses in adult *Ocm^−/−^* mice. In apical turn whole mounts, P2X2 receptors were found in the organ of Corti of both GCaMP6s + *Ocm^+/+^* and *Ocm^−/−^* mice. GCaMP6s + *Ocm^−/−^* but not *Ocm^+/+^* mice showed strong P2X2 receptor labeling in the OHC region, especially in supporting cells and near the cuticular plate, before noise exposure, demonstrating that the upregulation is maintained into adulthood (Fig. 8*A*). After prolonged noise exposures, GCaMP6s + *Ocm^+/+^* OHCs showed stronger P2X2 receptor immunolabeling in the stereocila bundles compared with prenoise *Ocm^+/+^* mice (Fig. 8*B*, red arrows). However, there was little change in P2X2 receptor immunolabeling between pre- and postnoise *Ocm^−/−^* OHCs.

Without noise exposure, the cochlear expression of *P2RX2* mRNA in 3–4-week-old *Ocm^−/−^* mice was significantly higher than that in GCaMP6s + *Ocm^+/+^* mice (*p* = 0.0036; Fig. 8*C*). After prolonged noise exposure, *P2RX2* expression was significantly upregulated in the GCaMP6s + *Ocm^+/+^* cochlea (*p* = 0.0054) but not in GCaMP6s + *Ocm^−/−^* cochlea (*p* = 0.3472; Fig. 8*C*). There was no significant difference in *P2RX2* expression between postnoise *Ocm^+/+^* and *Ocm^−/−^* OHCs (*p* = 0.9933, post hoc test two-way ANOVA; Fig. 8*C*). Western blot experiments also showed that prolonged noise stimulation significantly upregulated the expression of P2X2 receptors in *Ocm^+/+^* mice (*p* = 0.0395, post hoc test from two-way ANOVA; Fig. 8*D*). The expression of P2X2 receptor in pre- and postnoise *Ocm^−/−^* mice showed no significant difference (*p* = 0.9523, post hoc test from two-way ANOVA; Fig. 8*D*). These data suggest that the absence of OCM contributes to a sustained upregulation of P2X2 receptors in the organ of Corti that does not further respond to prolonged noise exposure.

## Discussion

In this study, we demonstrate that loss of the Ca^2+^ buffering protein OCM creates an inner ear with a noise-exposed phenotype that has increased vulnerability to further noise exposure, increased Ca^2+^ signaling responses, and altered expression of purinergic receptors in young adult mice. We used both short-duration (2 h) and long-duration (9 h) moderate noise exposures (95 dB SPL) to probe for differences in recovery and cumulative Ca^2+^ response. Specifically, sustained noise was used to maximize purinergic receptor expression (Wang et al., 2003). Although short-duration noise exposure caused similar acute shifts in ABR and DPOAE thresholds in *Ocm^+/+^* and *Ocm^−/−^* mice 6 h postexposure, only *Ocm^−/−^* mice had permanent shifts after 2 week recovery. Both before and after noise exposure, *Ocm^−/−^* mice exhibited more variable neural responses. Prolonged noise exposures (95 dB SPL, 9 h) elicited larger threshold shifts and increased ATP-induced Ca^2+^ signaling in *Ocm^−/−^* OHCs compared with *Ocm^+/+^* OHCs and increased expression of purinergic P2X2 receptors in the cochlea of *Ocm*^+/+^ but not in *Ocm*^−/−^ mice. Upregulated ATP-induced Ca^2+^ responses and maintained elevated levels of P2X2 in mature *Ocm^−/−^* OHCs suggest they may have a higher Ca^2+^ load (Murtha et al., 2022; Yang et al., 2023). Furthermore, *Ocm*^−/−^ mice showed modified Ca^2+^ responses to noise in IHCs and supporting cells, suggesting that alterations in OHC Ca^2+^ buffering lead to compensatory changes throughout the sensory epithelium. Together, these results indicate that chronic dysregulation of Ca^2+^ homeostasis in *Ocm*^−/−^ mice dramatically alters cochlear function and causes increased susceptibility to cochlear pathology that likely contributes to early-onset hearing loss in the *Ocm*^−/−^ mice.

Although ABR and DPOAE thresholds are sensitive to hair cell damage, they are relatively insensitive to synaptic loss or neural pathology (Kujawa and Liberman, 2009; Stamper and Johnson, 2015; Liberman and Kujawa, 2017). In our study, thresholds were similar between genotypes before exposure and equally shifted 6 h after noise exposure. However, ABR and DPOAE thresholds failed to recover in *Ocm^−/−^* mice as they did in *Ocm^+/+^* mice, suggesting sustained sensory cell damage or loss. Since prenoise thresholds were similar between the two genotypes, we examined the underlying neural coding. ABRs represent the synchronized activity of auditory nerve fibers contacting IHCs (Buchwald and Huang, 1975; Chen et al., 2006). Prior studies show that TTS can mask IHC synaptic loss (Lavinsky et al., 2015; Mehraei et al., 2016; Liberman and Kujawa, 2017). Unlike *Ocm^+/+^* mice, the *Ocm^−/−^* mice had variable wave I amplitudes, and longer wave I latencies both before and after noise exposure. These alterations did not reflect baseline synaptic deficits, as there was no decrease in synaptic counts in *Ocm^−/−^* mice prior to noise exposure. While studies have reported OHC ribbon changes after noise exposure (Wood et al., 2021), we found no significant genotype-dependent differences.

Our results suggest that OCM contributes to OHC homeostasis by supporting depolarization-activated currents and buffering intracellular Ca^2+^. Although the voltage-dependent properties of *Ocm*^−\−^ OHCs were broadly preserved, peak current amplitudes were reduced at positive potentials suggesting altered contributions from MET channels, voltage-gated Ca^2+^ channels, or other Ca^2+^-dependent channels augmented by Ca^2+^ buffering. The primary sources of Ca^2+^ entry into OHCs are MET channels at the tips of stereocilia and voltage-gated Ca^2+^ channels (Ca_V_1.3, *CACNA1D*) along the basolateral membrane (Knirsch et al., 2007; Pangrsic et al., 2018; Qiu and Muller, 2018). Disruption of any of the key proteins involved in OHC Ca^2+^ homeostasis leads to OHC dysfunction, loss, and subsequent hearing loss. Several studies report that deletion of Ca_V_1.3 leads to an early loss of OHCs (Platzer et al., 2000; Glueckert et al., 2003; Engel et al., 2006; Eckrich et al., 2019). In the present study, *Ocm* deletion led to significantly higher GCaMP6s + fluorescence in both IHCs and OHCs. Since GCaMP6s reports relative Ca^2+^ changes, increased GCaMP6s + fluorescence intensity could reflect altered buffering, slower decay kinetics, or greater spatial summation (Yang et al., 2023). We investigated whether this increased fluorescence was due to altered Ca_V_1.3 expression, which serves as one of the main Ca^2+^ entry points in hair cells (Platzer et al., 2000; Michna et al., 2003). We found that *Ocm* deletion had little to no effect on Ca_V_1.3 expression. Before noise, Ca_V_1.3 expression was similar in *Ocm*^+/+^ and *Ocm*^−/−^ mice. Following prolonged noise exposure, there were similar increases in Ca_V_1.3 expression in *Ocm*^+/+^ and *Ocm*^−/−^ mice. Since *Ca_v_1.3* mRNA is downregulated in prehearing *Ocm*^−/−^ mice (Yang et al., 2023), it was speculated that Ca_V_1.3 downregulation might be a response to prevent Ca^2+^ overloading in OHCs and thus protect them from early cytotoxicity. However in the present study, the lack of Ca_V_1.3 downregulation in young adult *Ocm*^−/−^ mice and the similar Ca_V_1.3 expression profiles before and after noise in both in *Ocm*^+/+^ and *Ocm*^−/−^ mice, suggest that Ca_V_1.3 plays less of a role in *Ocm*^−/−^ mice vulnerability to noise.

Mitochondria also play a key role in OHC Ca^2+^ regulation. Thus, OHC mitochondria could temporarily compensate for the absence of OCM and maintain OHC function. There is altered mitochondrial protein expression and increased OHC loss in *Ocm*^−/−^ mice as soon as 24–48 h after intense noise (106 dB SPL, 2 h; Murtha et al., 2024). The current findings indicate that even moderate noise produces exaggerated Ca^2+^ responses in *Ocm*^−/−^ OHCs, potentially overwhelming mitochondrial buffering capacity. Any dysfunction in Ca^2+^ buffering likely leads to greater sensitivity to noise as demonstrated by the present study but also OHC loss as demonstrated by Murtha et al. (2024). Thus, both OCM and mitochondria are likely to determine the ability of OHCs to compensate for large increases in Ca^2+^ after acoustic overexposure (Fettiplace and Nam, 2019). Furthermore, the presence of threshold shifts, despite the lack of OHC loss after moderate noise exposure, suggests widespread, intrinsic dysfunction of the OHC separate from common degenerative pathways such as apoptosis and necrosis. Studies using high-intensity noise exposure had suggested these threshold shifts were due to OHC loss, not intrinsic dysfunction (Murtha et al., 2024). Our findings accentuate the difference in OHC response to high-intensity versus moderate-intensity noise. This difference suggests hair cells may undergo intrinsic dysfunction prior to loss and that OCM may play a role in delaying cellular senescence.

In agreement with our study, others have reported that sustained noise exposure leads to an upregulation of *P2RX2* mRNA and protein levels in the organ of Corti (Wang et al., 2003; Telang et al., 2010). Sustained (6–24 h) moderate noise primarily induces a stable, protective cochlear adaptation dominated by sustained purinergic signaling and gain suppression. The high expression of purinergic receptors, with an increase in local ATP release in the endolymph and perilymph (Munoz et al., 1995; Telang et al., 2010), may provide a shunt capable of regulating the electrical potential across the endolylmphatic surface of hair cells (Thorne et al., 2004). The mutation of *P2RX2* in both humans and mice gives rise to progressive hearing loss and increased sensitivity to noise-induced PTS (Yan et al., 2013). Furthermore, a recent study revealed an increase in P2Y purinergic response in supporting cells from the aged cochlea (Hool et al., 2023), indicating a possible protective mechanism that prevents further damage to the sensory epithelium. In *Ocm*^−/−^ mice, upregulation of purinergic receptors could be a compensatory mechanism for Ca^2+^ overload. Blocking purinergic receptors with PPADS abolished genotype-dependent differences, confirming P2X2-mediated Ca^2+^ influx as a major contributor to the exaggerated Ca^2+^ signaling. Elevated purinergic signaling may initially be compensatory, but chronic upregulation can enhance inflammation and stress responses (Burnstock, 2016; Liu et al., 2023), ultimately worsening noise-induced cochlear pathology.

Our results further demonstrate the importance of OCM as a specialized Ca^2+^ buffer in OHCs that is essential for OHC function and survival and minimizes the consequences of noise-induced cochlear insults. The homeostatic control of intracellular Ca^2+^ likely contributes to the sensitivity of the OHCs to cochlear injury (Mammano, 2011). Aberrant Ca^2+^ dynamics in hair cells contribute to cochlear pathology (Richard et al., 2023) and increased OHC loss in *Ocm*^−/−^ mice (Tong et al., 2016; Climer et al., 2021). Noise exposure, a common cause of hearing loss, is associated with increased concentration of cytosolic free Ca^2+^ (Fridberger et al., 1998; Zuo et al., 2008), and finally, Ca^2+^ overload in sensory hair cells can activate regulated cell-death pathways, which, in turn, could exacerbate inflammatory responses (Fridberger et al., 1998; Bienkowski et al., 2000; Orrenius et al., 2003; Esterberg et al., 2013; Fu et al., 2021). Multiple studies suggest that Ca^2+^ signaling plays fundamental roles in cochlear development and function, including mechanotransduction, neurotransmitter release, innervation, and motility (Ceriani et al., 2025). Similarly, other studies have reported that *Ocm*^−/−^ OHCs show increased spontaneous Ca^2+^ activity, delayed synaptic maturation, and altered afferent innervation patterns during development (Yang et al., 2023).

In summary, our findings highlight OCM as an essential regulator of Ca^2+^ homeostasis in OHCs and a key determinant of vulnerability to acoustic injury. In the absence of OCM, Ca^2+^ homeostasis is significantly altered, creating an ear with a noise-exposed phenotype. We found that *Ocm^+/+^* OHCs respond to moderate noise by upregulating purinergic receptor expression and increasing intracellular Ca^2+^ signaling, leading to temporary changes in OHC depolarization, amplification, and ABR wave I amplitude and latency. In contrast, OHCs lacking OCM exhibit chronic dysregulation of Ca^2+^ represented by increased ATP-induced Ca^2+^ activity, higher expression of ATP receptors in OHCs, reduced depolarization-activated currents, and abnormal ABR wave I responses. Thus, adult *Ocm^−/−^* mice show a noise-exposed phenotype, which likely contributes to increased susceptibility to noise damage. We propose that OCM functions as a specialized Ca^2+^ buffer that protects OHCs from Ca^2+^ overload, thereby preserving cochlear function across development, adulthood, and environmental challenges such as noise. Furthermore, dysregulation of cytosolic Ca^2+^ homeostasis likely contributes to early-onset hearing loss in the *Ocm^−/−^* mice.

## Synthesis

Reviewing Editor: David Schoppik, New York University Grossman School of Medicine Decisions are customarily a result of the Reviewing Editor and the peer reviewers coming together and discussing their recommendations until a consensus is reached. When revisions are invited, a fact-based synthesis statement explaining their decision and outlining what is needed to prepare a revision will be listed below. The following reviewer(s) agreed to reveal their identity: NONE. Note: If this manuscript was transferred from JNeurosci and a decision was made to accept the manuscript without peer review, a synthesis may not be available.

The revised manuscript addresses the reviewers' concerns and is the stronger for the effort.
